# Effects of Intronic SNPs in the Myostatin Gene on Growth and Carcass Traits in Colored Polish Merino Sheep

**DOI:** 10.3390/genes11010002

**Published:** 2019-12-18

**Authors:** Ewa Grochowska, Bronisław Borys, Sławomir Mroczkowski

**Affiliations:** 1 Department of Animal Biotechnology and Genetics, UTP University of Science and Technology, Mazowiecka 28 St., 85-084 Bydgoszcz, Poland; mroslav@utp.edu.pl; 2 National Research Institute of Animal Production, Experimental Station Kołuda Wielka, Parkowa 1 St., 88-160 Janikowo, Poland; bronislaw.borys@onet.eu

**Keywords:** the myostatin gene (*MSTN*), sheep, growth, carcass, SNP, intronic polymorphism

## Abstract

Myostatin acts as a negative regulator of muscle growth; therefore, its role is important with regard to animal growth and meat production. This study was undertaken with the objective to detect polymorphisms in the first intron and c.*1232 position of the *MSTN* gene and to analyze effects of the detected alleles/genotypes on growth and carcass traits in Colored Polish Merino sheep. In total, 23 traits were analyzed, i.e., seven describing lamb growth and 16 carcass traits. Single nucleotide polymorphisms (SNPs) in the first intron and the c.*1232 position were identified using polymerase chain reaction single-strand conformation polymorphism (PCR-SSCP) and PCR-restriction fragment length polymorphism (PCR-RFLP) methods, respectively. The MIXED procedure of the SAS software package was used to analyze allelic and genotypic effects of the *MSTN* gene on growth and carcass traits. Polymorphisms were only detected in the first intron of the *MSTN* gene. All investigated sheep were monomorphic G in the c.*1232 position. The *MSTN* genotype was found to have significant effect on body weight at 2nd day of life (BW2) and loin and fore shank weights. Significant allelic effects were detected with respect to BW2, scrag, leg, fore, and hind shank weights. These results suggest that polymorphisms in the first intron of the *MSTN* gene are relevant with respect to several carcass traits and BW2 in Colored Polish Merino sheep.

## 1. Introduction

Constant improvement of lamb growth and meat yield of lamb carcasses is of high interest for sheep breeders due to direct benefits from breeding of faster growing animals with higher final body weight as well as carcass weight. Nowadays, sheep breeding is supported by various molecular genetics tools. One of them is a marker-assisted selection, which seems to be convenient choice, especially for less numerous indigenous sheep populations. This relatively cheap approach has a potential to increase rates of genetic gain for certain growth and carcass traits in different sheep populations.

Among many genes, which are involved in animal growth and which affect final carcass weight, the one coding for myostatin is an excellent candidate for the marker-assisted selection due to its crucial role in muscle development and growth. Indeed, myostatin, being a member of the transforming growth factor-β super-family, acts as a negative regulator of muscle growth. This protein is a homodimer, which is primary synthesized in skeletal muscle as a 375-amino acid propeptide. Subsequently, this propeptide is proteolytically processed at the RSRR (263) site to give rise to a 26-kDa active processed peptide, which binds to receptor to elicit biological function [1,2].

The myostatin gene (*MSTN*) is expressed both in developing and adult skeletal muscles. It is located on the second chromosome in sheep and consists of three coding exons. Whole gene length is 6757 bps (GenBank no NC_040253.1), whereas transcript consists of 1128 bps and codes for a protein with 375 amino acids in its final configuration [3] (Oar_v3.1, database version 97.31). In general, polymorphisms, especially those nonsynonymous ones, in coding regions of different genes, are of great importance due to their typically significant effect, positive or negative, on certain organism’s phenotype. For instance, in double-muscled cattle breeds, especially Piedmontese and Belgian Blue, a missense mutation or an 11 nucleotide deletion, respectively, in the third exon of the *MSTN* gene, inhibits production of mature, functional myostatin leading to increase in muscle mass caused by enhanced muscle development [4]. In sheep, Boman et al. [5,6] have detected single-base deletion c.960delG in coding region of the *MSTN* gene only in Norwegian White sheep, which resulted in completely non-functional protein. Additionally, Boman and Våge [7] found one base-pair insertion in coding part of this gene, which creates premature stop codon in the 49th amino acid position in protein exclusively in Norwegian Spælsau sheep. They ([5,6,7]) concluded that these two Indels positively affect sheep carcass traits in terms of increased muscularity and reduced fatness in investigated sheep breeds in Norway. The synonymous G/C transversion in the third exon of the *MSTN* gene was identified in two Indian sheep breeds [8], whereas the c.101A>G SNP (rs417816017) in the first exon was found in selected sheep breeds in China and New Zealand [9,10,11]. Moreover, Trukhachev et al. [12] have identified two more sequence variations in coding region of this gene, i.e., a single nucleotide insertion (c.782_783insT) and a new SNP c.940G>T in the third exon exclusively in Stavropol Merino sheep. However, in all beforementioned studies the association analyses of the effects of these SNPs on carcass traits in sheep were not undertaken.

Clop et al. [13] have found the G/A substitution in 3′UTR region of the *MSTN* gene that creates a target site for two microRNAs, i.e., mir1 and mir206, which are highly expressed in skeletal muscle. They also reported that this mutation results in muscular hypertrophy in Texel sheep due to translational inhibition of the myostatin gene. Indeed, it is well known that variants in non-coding regions of the genome can influence gene regulation and affect the phenotype. Other authors, e.g., [14,15], also confirmed effects of SNPs located in non-coding regions of the *MSTN* gene on growth, carcass and meat quality traits in sheep. For instance, Gan et al. [14], who investigated polymorphisms in a promoter, 5′UTR and 3′UTR regions as well as introns of the *MSTN* gene, identified 12 haplotypes. Notably, two of these haplotypes were significantly associated with certain growth traits in sheep. Kijas et al. [15] found a number of effects of SNPs located in the promoter, the second intron, and the 3′UTR regions on several carcass and meat quality traits. Moreover, Wang et al. [16] identified polymorphisms in the promoter region of the *MSTN* gene and showed their effects on growth and carcass traits in NZ Romney sheep.

Interestingly, Hickford et al. [17] detected polymorphisms in the first intron of the *MSTN* gene and reported associations between these alleles and a number of carcass traits in New Zealand Romney sheep. Sjakste et al. [18], who also identified a number of SNPs in the same fragment of the *MSTN* gene in Latvian Darkhead sheep, reported several reasons that suggested the polymorphisms in this non-coding region possess functionality as regulatory elements. For instance, the G/T transversion in the c.373+18 position could be functionally active affecting transcript splicing. Moreover, due to T nucleotide the transcription repressor AREB6 binding site could be created in the c.373+101 position [18]. Interestingly, the simultaneous analysis of substitutions in the positions c.373+241, 243, 246, 249, and 259, revealed four transcription factor binding sites (TFBSs) [18]. Sjakste et al. [18] also showed that in the majority of alleles the presence of cytosine in the c.373+323 position resulted in formation of the binding sites on the minus strand to both SMAD proteins, involved in muscle mass regulation in adulthood [19], and the MEIS1 cofactor of TALE (*HOX*) family, critical for many aspects of animal morphogenesis (reviewed by [20]).

Despite the abovementioned potential significant functionality of the SNPs located in the first intron of the *MSTN* gene, very limited number of association studies between these polymorphisms and growth as well as carcass traits in sheep have been undertaken. The majority of studies have focused on the G/A substitution in the 3′UTR region (c.*1232); however, this substitution has not been found in Merino sheep [14,15] despite having been reported in several other sheep breeds including New Zealand Romney [21]. For this reason, the present study was performed with the aim of identifying alleles and genotypes in the first intron of the *MSTN* gene and to estimate their effects on growth and carcass traits in Colored Polish Merino sheep. The presence/absence of the G/A transition in the c.*1232 position of this gene was also investigated. With respect to carcass traits, the novelty in this study was the first extensive analysis of allelic and genotypic effects in the first intron of *MSTN* gene on lamb carcass traits, involving weights of 10 different meat cuts, weights of 3 parts of a carcass as well as tissue composition of a leg. The importance of this study lies also in comprehensive analysis of association between polymorphisms in the first intron of the *MSTN* and growth traits in Merino sheep based on first results obtained in Colored Polish Merino sheep.

## 2. Materials and Methods

### 2.1. Animals

The studies were carried out on 264 purebred Colored Polish Merino lambs of both sexes, which were produced over three experimental years on a farm owned by the National Research Institute of Animal Production (NRIAP) Experimental Station Kołuda Wielka (Poland). Lambs were sired by nine different rams. Each sire was used for breeding during two or three experimental years. Sheep were raised indoors and at the pasture, where they grazed six times a week. Suckling lambs were fed dry, granulated mash and a meadow hay ad libitum.

The Colored Polish Merino is a variety of the Polish Merino sheep breed, which was produced by selecting the Polish Merino sheep for colored wool. Similarly to the Polish Merino sheep, the Colored Polish Merino sheep are used both for their meat and wool. Adult ewes and rams weight on average 55–65 kg and 80–100 kg, respectively [22]. Prolificacy of Colored Polish Merino ewes is 135% on average.

All procedures that involved animals were approved by the Local Animal Research Ethics Committee and the Local Veterinary Service.

### 2.2. Growth Traits

Growth data regarding body weights at second (BW2), 30th (BW30), 56th (BW56), and 78th (BW78) day of life were collected for 264 male and female lambs. Based on these data the average daily gains between second and 30th (ADG2-30), 30th and 56th (ADG30-56), as well as 56th and 78th (ADG56-78) were calculated for each individual under investigation. Moreover, birth rank and sex were recorded for each lamb at birth.

### 2.3. Carcass Traits

In total, 106 ram lambs at mean age 105 days (SD 4.2, range 92–119 days) were chosen for slaughter during the three years of the study. Slaughtering, carcass cutting, and leg dissection were done in the abattoir of the NRIAP Experimental Station Kołuda Wielka as described previously by Grochowska et al. [23,24] according to the methodologies of Nawara et al. [25] and Krupiński et al. [26]. In brief, the slaughtering of ram lambs took place in batches, three or four times a year from March to April. Lambs were electrically stunned, exsanguinated, and skinned. Carcasses were cooled down in a chilling room (~4 °C for 18 h). Thereafter, they were halved and weighted. The right-half carcass was divided into three parts, i.e., fore-part of the carcass, the full loin part, and the leg part. Each of these parts was weighed and further divided into the following cuts: Scrag, middle neck, shoulder, breast and flank, rib, loin, tenderloin, leg, fore shank, and hind shank according to the methodology of Nawara et al. [25]. All cuts were weighted. The leg was dissected for three tissues: Muscles, bones, and fat, which were weighted separately and a yield of each tissue in the leg was calculated.

### 2.4. Genotyping of the Ovine MSTN Gene

DNA extraction and the identification of the *MSTN* gene polymorphisms were performed on 264 sheep as described previously by Grochowska et al. [24]. Briefly, total genomic DNA was purified from the whole blood using a MasterPure^TM^ DNA Purification Kit for Blood Version II (Epicentre, Madison, WI, USA). Polymorphisms in the first intron as well as the c.*1232 position, that is located in the 3′UTR region, of the *MSTN* gene were analyzed. Genotypes in the first intron were identified using the PCR-SSCP method according to the methodology of Hickford et al. [17] with later modifications implemented by Grochowska et al. [24]. Thereafter, PCR products representative of unique SSCP banding patterns were cleaned up using the ExoSAP-IT^®^ (Affymetrix, Santa Clara, CA, USA) and sequenced in both directions in Genomed, Poland. Moreover, the A/G substitution at the c.*1232 position of the ovine *MSTN* gene was detected using PCR-RFLP method as presented by Clop et al. [13] with later modifications described by Grochowska et al. [24].

### 2.5. Transcription Factor Binding Sites (TFBSs) Prediction

The AnimalTFDB (version v3.0) (http://bioinfo.life.hust.edu.cn/AnimalTFDB/) [27] was used to predict changes of the TFBSs that were caused by the identified SNPs in the first intron of the *MSTN* gene in sheep.

### 2.6. Statistical Analyses

Allele and genotype frequencies in the *MSTN* locus were calculated. Moreover, the observed and expected heterozygosity as well as the Hardy–Weinberg equilibrium test calculations were performed in Arlequin 3.5.1.2 [28].

SAS software package [29] (SAS Institute, Cary, NC, USA) was used to analyze the dataset. The effects of the *MSTN* genotypes or a number of copies of a certain *MSTN* allele on growth traits were estimated using the MIXED procedure. The following mixed-effect model was applied:

Y_ijkl_ = μ + a_i_ + b_j_ + c_k_ + d_l_ + f_m_+ e_ijklm_ (1)

where Y_ijkl_ is the performance of the nth individual lamb for each trait of interest, μ is the general mean for each trait of interest, a_i_ is the fixed effect of the ith *MSTN* genotype (i = *MSTN*-A/*MSTN*-A, *MSTN*-A/*MSTN*-C, *MSTN*-A/*MSTN*-E, and *MSTN*-A/*MSTN*-E1) or the fixed effect of the ith number of copies of a certain *MSTN* allele (i = 0 or 1 for the *MSTN*-C, *MSTN*-E, and *MSTN*-E1 allele or i = 1 or 2 only for the *MSTN*-A allele), b_j_ is the fixed effect of the jth litter size (j = 1 (single), 2 (twin)), c_k_ is the fixed effect of the kth year of observation (k = 2011, 2012, 2013), d_l_ is the fixed effect of the lth sex (l = male, female), f_m_ is the random effect of the mth sire (m = sire 1, 2, …, 9), and e_ijklm_ is the random error.

Relationship between the *MSTN* genotype or a number of copies of a certain *MSTN* allele and carcass traits were analyzed using the model described above without the effect of the sex because only male lambs were investigated. In addition, the right-half carcass weight was found to be significant; therefore, it was fitted in the final model as the covariate with exception of three traits i.e., muscle, fat and bone tissue yields.

With regard to aforementioned models, for all variables, main effects and two-way interactions between fixed effects were tested. Generally, these interactions did not show a significant effects on the majority of analyzed traits; therefore, only single significant interactions were fitted in the models exclusively for selected traits although not for the others. Moreover, once the *MSTN* genotype or a number of copies of a certain *MSTN* allele was found to be statistically significant in the abovementioned models for certain traits, the significance of deviations was tested using the Tukey–Kramer test.

Analyses of relationships between the *MSTN* genotypes and growth traits covered sheep with genotypes that fulfilled the criterium of minimal sample size (n = 10; i.e., *MSTN*-A/*MSTN*-A, *MSTN*-A/*MSTN*-C, *MSTN*-A/*MSTN*-E, and *MSTN*-A/*MSTN*-E1). With regard to carcass traits, which were recorded for male lambs, only two genotypes excided the sample size of 10, i.e., *MSTN*-A/*MSTN*-A and *MSTN*-A/*MSTN*-E. Effects of a number of copies of a certain *MSTN* allele on growth traits were estimated separately for each of four alleles (i.e., *MSTN*-A, *MSTN*-C, *MSTN*-E and *MSTN*-E1), which fulfilled the criterium of minimal sample size (n = 10). With respect to carcass traits only three alleles, i.e., *MSTN*-A, *MSTN*-E, and *MSTN*-E1 reached the sample size of at least n = 10; therefore, allelic effects were tested only for them.

## 3. Results

### 3.1. Detected MSTN Alleles and Genotypes and Their Frequencies

**Figure 1:**
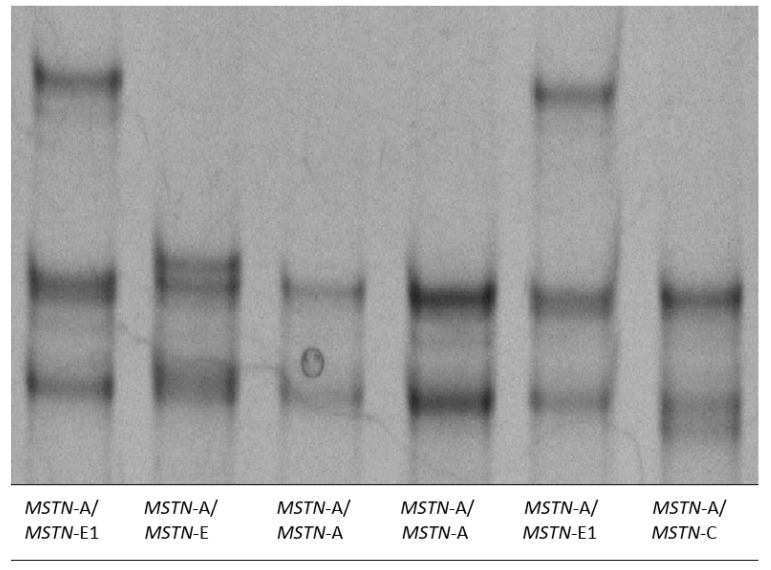
PCR-SSCP conformational polymorphism in the first intron of the ovine *MSTN* gene. Representative SSCP patterns for the four unique alleles (*MSTN*-A, *MSTN*-C, *MSTN-E*, and *MSTN*-E1) in both homozygous and heterozygous genotypes are shown.

In the studied Colored Polish Merino sheep, the SSCP analysis of polymorphisms in the first intron of the *MSTN* gene revealed four alleles, i.e., *MSTN*-A, *MSTN*-C, *MSTN*-E, and *MSTN*-E1, and four genotypes (Figure 1), which were previously described by Hickford et al. [17] and/or Grochowska et al. [24]. Polymorphisms of these four alleles as well as frequencies of these alleles and genotypes are shown in Table 1 and Table 2, respectively. The *MSTN*-A allele was predominant (87.7%), whereas the *MSTN*-C allele occurred with the lowest frequency of 2.3%. Consequently, the *MSTN*-A/*MSTN*-A homozygotes were the most frequent (75.4%) individuals in the investigated flock, while *MSTN*-A/*MSTN*-C sheep were the rarest ones (4.5%). With the regard to polymorphisms in the first intron of the *MSTN* gene, the value of observed heterozygosity (Ho = 0.25) was found to be slightly higher than the value of expected heterozygosity (He = 0.23). The population was in the Hardy–Weinberg equilibrium (*p* = 0.83).

**Figure 2:**
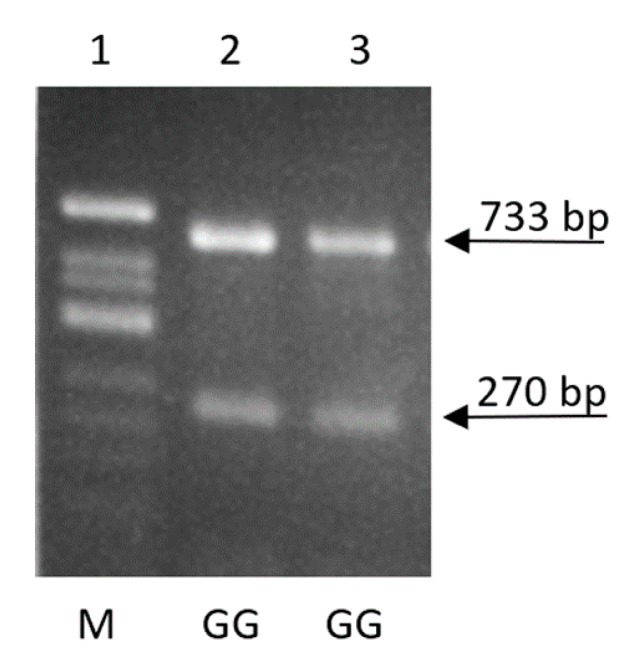
Result of PCR-RFLP analysis of the c.*1232 position in the 3′UTR of the *MSTN* gene in Colored Polish Merino sheep. A 1003-bp DNA fragment, including the c.*1232G>A SNP, was amplified by PCR, digested using *Hpy*CH4IV (New England Biolabs, Ipswich, MA, USA) restriction enzyme and separated electrophoretically on 2% agarose gel. The *Hpy*CH4IV endonuclease recognizes restriction site in G allele and cleaves the PCR product into a 270-bp and a 733-bp fragment. Line 1—a molecular-weight size marker pBR322/*Alu*I (Fermentas, Vilnius, Lithuania); line 2 and 3—homozygotes GG.

All investigated animals were GG homozygotes with respect to the c.*1232 SNP position of the *MSTN* gene (Figure 2). The A allele, which is associated with muscular hypertrophy in sheep [13], was absent in the studied sheep.

### 3.2. Transcription Factor Binding Sites (TFBSs)

The variation of TFBSs caused by six SNPs in the investigated fragment of the first intron of the *MSTN* gene was predicted using AnimalTFDB software. The G allele in the c.373+18 position created the putative binding sites for the following transcription factors-SETDB1, ESR1, ESRRA, and NRIP1, whereas the T allele created the putative binding sites for transcription factor RFX1, RFX2, and SETDB1. It was observed that the binding site for transcription factor GMEB1 was created due to both T and C allele in the c.373+18 position of the *MSTN* gene. Furthermore, the binding sites for the transcription factors SOX7, SOX21, and SMAD4 were created because of the A allele in the c.373+243 position. The binding site for transcription factor DMRT1 was created due to the simultaneous presence of the A and C alleles in the c.373+243 and c.373+249 positions. The C allele in the c.373+249 position was predicted to create the binding site for the transcription factor GTF2B, whereas the T allele in this position resulted in the creation of the binding site for transcription factor TRIM28. Moreover, the simultaneous presence of C and T allele in the c.373+249 and c.373+249 position was shown to create the binding site for the transcription factor POU6F1. It was predicted that the T allele in the c.373+259 position created the putative binding site for the transcription factor ESR1. The binding site for transcription factor BRD4 was created due to the T or C allele in the c.373+323 position. Moreover, the C allele in this position resulted in the creation of the binding sites for transcription factors OSR1 and POLR2A.

### 3.3. Effects of MSTN Alleles and Genotypes on Growth Traits

Results of the association analyses of *MSTN* genotypes and alleles with growth traits in Colored Polish Merino sheep are presented in Table 3 and Table 4, respectively. The general linear mixed model analyses showed significant effect of the *MSTN* genotype only on BW2 (*p* = 0.042). The *MSTN*-A/*MSTN*-E1 heterozygous lambs were on average 10% heavier than homozygotes *MSTN*-A/*MSTN*-A. With regard to BW2 it was found that this difference was due to effect the *MSTN*-E1 allele, but was not related with the number of copies of the *MSTN*-A allele. The presence of the *MSTN*-E1 allele was highly significantly related (*p* = 0.006) with higher BW2, whereas the *MSTN*-A allele was not associated with this trait (Table 4). Assessment of the allelic effects of the *MSTN* gene on the other growth traits did not show any significant associations.

### 3.4. Effects of MSTN Alleles and Genotypes on Slaughter Traits

Table 5 shows the results of association analyses between the *MSTN* genotypes and slaughter traits in Colored Polish Merino sheep, whereas Table 6 presents the effects of a number of copies of *MSTN* alleles on these traits. Among the 16 traits that were studied, weights of the two meat cuts, i.e., loin and fore shank, were significantly and highly significantly, respectively, affected by the *MSTN* genotype (*p* = 0.025 and *p* = 0.0018, respectively). *MSTN*-A/*MSTN*-E lambs had 6.2% heavier fore shank cuts when compared with *MSTN*-A/*MSTN*-A (Table 5). With respect to this trait, performed statistical analysis revealed highly significant (*p* = 0.005) effect of a number of copies of the *MSTN*-A allele, whereas animals carrying one copy of this allele had heavier fore shank cut (3.9%) than those ones with two copies. In contrast, the presence of the *MSTN*-E allele in the lamb genotype resulted in highly significantly (*p* = 0.009) higher fore shank weight when compared with counterparts without this allelomorph (Table 6). On the other hand, *MSTN*-A homozygotes had 6.6% heavier loin cut (Table 5) than the *MSTN*-A/*MSTN*-E heterozygotes. Analysis of the effect of a number of copies of the *MSTN*-A allele on this trait confirmed that an additional copy of this allele positively affects loin cut weight (*p* = 0.053; Table 6). Additionally, the results of the association analysis between a number of copies of *MSTN* alleles and carcass traits indicated significant negative effect of the additional copy of the *MSTN*-A allele on scrag and leg weight. Interestingly, lambs harboring one copy of the *MSTN*-E allele had 3.8% heavier hind shank cut (*p* = 0.027) when compared with lambs without this allele (Table 6).

## 4. Discussion

In this study we presented allelic and genotypic effects of the *MSTN* gene (first intron) on economically important traits in lamb production, i.e., growth traits and meat cuts weights. To our knowledge, it is the first so extensive study of the associations between polymorphism in the first intron of the *MSTN* gene and growth traits as well as meat cuts weights in a merino sheep breed not only in Europe, but also worldwide. The better understanding of the impact of polymorphisms in the first intron of the *MSTN* gene on growth and carcass, especially in sheep breeds lacking G/A transition in the c.*1232 position, is of high interest due to its potential effect on lamb production. Importantly, significant associations between alleles and/or genotypes in this region of the *MSTN* gene and growth as well as carcass traits, could offer the potential to improve meat production in sheep breeds without beforementioned substitution in the 3′UTR region.

### 4.1. Detected MSTN Alleles and Genotypes and their Frequencies

Our present and previous [24] study of the *MSTN* gene polymorphisms, together with analyses performed by Kijas et al. [15], showed that the investigated sheep of a merino breed, do not possess a loss of function SNP in the 3′UTR (the c.*1232A allele). For this reason, we focused our attention on polymorphisms in the first intron of the *MSTN* gene. Other authors, e.g., [13,14,17,18], reported that this region of the ovine *MSTN* gene was highly polymorphic in different sheep breeds. Indeed, we confirmed these observations in Colored Polish Merino sheep in our present and previous study [24] detecting four and five different alleles, respectively. Furthermore, detecting 5 alleles and 7 genotypes, we also recognized that other two sheep breeds in Poland, i.e., Kamieniec and Pomeranian, are even more polymorphic with respect to the first intron of the *MSTN* gene [30]. Interestingly, Sjakste et al. [18] indicated several reasons suggesting that the SNPs located in the non-coding region of the *MSTN* gene, i.e., the first intron, could have functionality as regulatory elements. For instance, the G nucleotide in the c.373+18 position, which is only present in the *MSTN*-A allele in Colored Polish Merino sheep, initiates processes that could potentially increase the transcription and splicing efficiency of the c.373+18G sequence variant compared to the c.373+18T alleles (i.e., the *MSTN*-C, *MSTN*-E, and *MSTN*-E1 alleles in the present study). Consequently, Sjakste et al. [18] concluded that the c.373+18T variant should be considered as advantageous in meat breeding programs in sheep. Additionally, the T nucleotide in the c.373+101 position, which we detected only in the new *MSTN*-E1 allele [24], creates the transcription repressor AREB6 binding site. In contrast, the cytosine in this position, which is more common in a number of sheep breeds [13] as well as Colored Polish Merino sheep [24], could potentially change sequence bendability following the formation of the DNA curvature [18]. With regard to substitutions in the positions c.373+241, 243, 246, 249, and 259, Sjakste et al. [18] deducted that the Hap 1 = A sequence variant can specifically bind those transcription factors (TFs) that potentially negatively influence myogenesis. In the present study, all detected alleles with only one exception of the *MSTN*-C allele have cytosine in the c.373+323. Sjakste et al. [18] found that in the majority of alleles cytosine in this position defines the minus strand affinity to SMAD proteins and the MEIS1 cofactor of TALE (*HOX*) family. SMAD proteins are involved in muscle mass regulation in adulthood [19]. The MEIS1 cofactor of TALE (*HOX*) family is critical for many aspects of animal morphogenesis (reviewed by [20]). On the other hand, thymine in the c.373+323 position (the *MSTN*-C allele in our studies) generates new GATA1 site on the plus strand and controls the affinity to the nonspecific transcriptional activator of the GATA family involved in muscle growth regulation [31].

In the preset study, the *MSTN*-A allele and consequently the *MSTN*-A/*MSTN*-A genotype were the most frequent ones in Colored Polish Merino sheep. We reported similar results in our previous study [24]. To our knowledge, there have not been other studies than ours regarding allelic and genotypic frequencies in the first intron of the *MSTN* gene in a Merino sheep; therefore, it is hard to compare them with other studies in Merino sheep worldwide. Only Hickford et al. [17] reported the presence of five alleles, i.e., A, B, C, D, and E, detected in Merino & Polwarth in New Zealand. Other authors, i.e., Clop et al. [13], Gan et al. [14], Trukhachev et al. [12,32] provided information about polymorphic positions in 5 Merinos Arles sheep in France, two Merino breeds in China, Stavropol Merino and New Zealand Merino, and 30 Dzhalginsky Merino rams in Russia, respectively, but they did not classified these SNPs into alleles or genotypes.

With respect to the first intron, we observed considerably lower frequency of the *MSTN*-A allele (51.1%) in Kamieniec sheep breed and higher occurrence of this allele in Pomeranian sheep breed in Poland [30] as compared with Colored Polish Merino in the present study. Interestingly, in Kamieniec sheep the *MSTN*-B allele, not detected in the preset study, occurred with the frequency of 46.5%. On the other hand, Farhadian et al. [33] observed lower percentage of the A allele (42%) together with higher occurrence of the C and D alleles (23% and 21%, respectively) in Makoei sheep in Iran as compared to Colored Polish Merino, Kamieniec, and Pomeranian sheep breeds in Poland. Based on the results of the present study as well as other abovementioned ones, it could be concluded that the first intron of the *MSTN* gene is polymorphic, whereas both the number of alleles/haplotypes as well as their frequencies vary with respect to sheep breed, its purpose, and region of an origin. Moreover, breeding history together with genetic background of the breed play important roles in modulating the polymorphism in the first intron of the *MSTN* gene. Considering the fact, that a great number of sheep breeds from all around the world have not been investigated in terms of polymorphism in the first intron of the *MSTN* gene, information about nucleotide variations in this part of the *MSTN* gene in different sheep breeds all around the world could be very valuable.

### 4.2. Effects of MSTN Alleles and Genotypes on Growth Traits

Myostatin acts as a negative regulator of muscle growth. Growth traits are of great interest for breeders because they influence the profit form lamb production. It is well known, that polymorphisms in non-coding regions can have a significant effect on a regulation of the gene expression. Therefore, they can finally affect a phenotype. Indeed, in the current study we showed effect of intronic polymorphisms on one out of seven analyzed growth traits, i.e., BW2. We found that *MSTN*-A/*MSTN*-E1 heterozygous lambs were significantly heavier on the second day of life than *MSTN*-A/*MSTN*-A homozygotes. Subsequent analysis of the allelic effect showed that this difference was due to effect of the *MSTN*-E1 allele. Similarly, Farhadian et al. [33] found the effect of polymorphisms in the first intron of the *MSTN* gene on birth weight, but not on other growth traits, in Iranian Makoei sheep. Additionally, our previous results showed both genotypic and/or allelic effects of the *MSTN* intronic polymorphisms on several carcass and meat quality traits in Colored Polish Merino sheep [24] as well as growth traits in Kamieniec sheep breed [30]. In contrast, Hickford et al. [17] did not find any association between polymorphisms in the first intron of the *MSTN* gene and growth traits in New Zealand Romney sheep.

Several authors have also focused on the effects of nucleotide variations in other non-coding regions of the *MSTN* gene than the first intron [15,16,21,34,35,36,37] or/and polymorphisms in coding region of this gene [6,8,10] on growth traits. For instance, Kijas et al. [15] analyzed SNPs in the promoter region, the second intron and the c.*1232 position of the *MSTN* gene; however, they did not find any relationships between these nucleotide substitutions and several lamb growth traits, e.g., birth weight, in different sheep breeds. These results partially correspond with findings of Masri et al. [35] and Johnson et al. [38], who also did not show effects of the c.*1232G>A transition (formerly g+6723G>A) on several growth traits. On the other hand, we found effect of this SNP on body weight at 56th day of life and average daily gain in Kamieniec sheep breed in Poland [30]. Moreover, the c.*1232G>A substitution was associated with lamb live weight in 8 weeks of age in Texel x Welsh Mountain lambs [36]. Interestingly, Wang et al. [16] found association between the nucleotide variation in a promoter region of the *MSTN* gene (c.-2379) and birth, tailing and weaning weights. Moreover, Han et al. [37] investigated SNPs in the promoter, the first exon and intron, as well as the 3′UTR region of the *MSTN* gene and they observed highly significant (*p* = 0.006) and significant (*p* = 0.02) effects of the H1 haplotype on tailing weigh (approximately 3 weeks after birth) and growth rate to weaning, respectively.

SNPs in coding regions are even of higher interest than those ones in non-coding fragments of the gene due to their direct effects on the structure of a protein. According to Ensembl database there are 8 SNPs in coding region of the ovine *MSTN* [3] (Oar_v3.1, database version 98.31), whereas only 3 of them are synonymous variants. However, there are sparse information on associations between these polymorphisms and production traits in sheep mostly due to the fact that they have been detected in single sheep breeds. For instance, Ma et al. [10] showed relationship between SNP in the first exon of the *MSTN* gene (rs417816017) and several growth traits, among them birth weight, in Tan sheep. Boman et al. [6] found an effect of a deletion in coding region of the *MSTN* gene (c.960delG) on weaning weight and live weight gain in Norwegian White Sheep. Ranjan et al. [8] reported an association of the SNP in the third exon of the *MSTN* gene with body weights in 9 and 12 months old Madras Red sheep, whereas birth weight was not affected by this SNP.

As mentioned before, in the present study the *MSTN*-E1 allele affected significantly BW2, but not other growth traits under investigation. This result may suggest that the *MSTN*-E1 allele plays a role rather in prenatal muscle development than in postnatal growth in Colored Polish Merino sheep. Interestingly, Casas et al. [39] and Short et al. [40] observed an association between *MSTN* gene mutations and calving difficulties in cattle. However, Crispo et al. [41], who studied fetal and postnatal development and delivery in wild type (WT) lambs and their *MSTN* knock-out (KO) counterparts (using CRISPR/Cas9 technology), did not find evidence on the effect of the myostatin gene on prenatal lamb growth. They observed that homozygous *MSTN* KO lambs were not significantly different than WT animals in terms of body weight at birth. Moreover, conversely to our results Crispo et al. [41] and Li et al. [42] showed significant differences between the WT and the *MSTN* KO lambs in postnatal growth and development. Results of Crispo et al. [41] are with disagreement not only with our present studies, but also with outcomes of other authors, e.g., [10,16,34], who have observed the effects of polymorphisms in the *MSTN* gene on birth weight. Conversely, several authors, e.g., [15,38], did not observe effects of the *MSTN* gene polymorphisms on postnatal growth. In the *MSTN* KO lambs the myostatin is completely inactive; therefore, the effects are more profound when compared them to lambs with different *MSTN* genotypes. Lambs having the *MSTN* gene in their genomes differ in terms of genotype in the locus of this gene and the differences between genotype groups are often not significant due to functions of other gene fragments, which simultaneously play a role in gene function and expression.

Taking into consideration both our results and those reported by other authors, it could be concluded that the effect of the *MSTN* gene polymorphisms on growth traits vary on type of polymorphism and sheep breed. Like many other production traits, growth characteristics belong to complex traits, which are affected by polygenes as well as environmental factors. Consequently, different results of association analyses obtained for certain sheep breeds are probably due to divergent genetic background of the breeds of interests, which may affect the magnitude and, perhaps, even the mode of action of the *MSTN* gene effects on lamb growth [43]. Additionally, environmental factors, e.g., feeding, are of great importance with respect to animal growth. Therefore, it could be assumed that both breeding history and environmental effects, together with the genetic background of the sheep breed, play roles in modulating the effects of selected SNP in the *MSTN* gene on lamb growth. Moreover, different results of the aforementioned association analyses are probably caused by differences in the *MSTN* gene polymorphism as well as in allele/haplotype frequencies between investigated sheep breeds in the present study as well as studies of other authors.

### 4.3. Effects of MSTN Alleles and Genotypes on Slaughter Traits

Polymorphisms in the *MSTN* gene are associated with the meatiness and fatness of lambs (e.g., [5,13,24]). Higher yield of lean meat is of high interest, as it guarantee breeder higher profit from lamb production. In the present study, we investigated genotypic and allelic effects of the *MSTN* gene on several lamb carcass cuts weights as well as tissue composition of a leg in Colored Polish Merino sheep. To our knowledge our analyses are the first attempt of assessing the relationships between the *MSTN* gene polymorphisms and abovementioned traits in Merino sheep. Additionally, for the first time we have reported the association of genotypes and alleles in the first intron of the *MSTN* gene with lamb carcass cuts weights in Merino sheep in Europe.

We observed that *MSTN*-A/*MSTN*-A homozygous lambs had 6.6% higher weight of one of the most valuable cuts, i.e., loin. Despite the fact that the effect of this genotype was not very large, on average 32 g, with regard to the weight of whole carcass, it could be relevant for a breeder in terms of profit from lamb production when considering a whole flock. As it is the first report on the association between alleles and genotypes in the first intron of the *MSTN* gene and lamb carcass cuts weighs both in Merino sheep and European sheep, it is hard to compare our results with those of other authors. To our knowledge only Hickford et al. [17] have investigated the associations between polymorphisms in the first intron of the *MSTN* gene and yields of three lamb carcass cuts, i.e., leg, loin and shoulder; however, the research was performed in New Zealand on Romney sheep. Despite the fact that Hickford et al. [17] conducted analyses on different sheep breed raised in divergent environmental conditions, their results are in partial agreement with those obtained by us in the current study. They also found negative effect of the A allele (comparable to the *MSTN*-A allele; see [24]) on leg yield. However, in contrast to our results, Hickford et al. [17] observed negative influence of this allele on the loin yield. Furthermore, Han et al. [37], who investigated association between the *MSTN* gene haplotypes and various carcass traits, showed that the presence of the H1 haplotype increased yields of such as valuable cuts as loin and leg as well as total yield. The H1 haplotype is similar to the *MSTN*-A allele detected in present study. Interestingly, Han et al. [37] reported that the presence of other haplotype, i.e., the H2 one, caused decrease in loin and leg yields, whereas for loin yield its effect was not significant. Remarkable, the H2 haplotype is also partially similar the *MSTN*-A allele from our current study (see [24]) in terms of polymorphism in the first intron and the c.*1232 position, and it differs from the beforementioned H1 haplotype only in the position the c.*1232 of the *MSTN* gene having A allele instead of G. This arrangement is in agreement with nucleotide variation encountered in Colored Polish Merino and other Merino sheep breeds, which do not possess the c.*1232 A allele [15,24]. These results suggest that the negative effect of the *MSTN*-A allele on leg weigh or yield could be similar in different sheep breeds raised in divergent environments, what may drove to conclusion that, with respect to this trait, genetic effect could be more relevant than environment.

Moreover, in the present study we observed that *MSTN*-A/*MSTN*-E heterozygotes had significantly heavier fore shank cuts (6.2%). Subsequent analysis of the effect of the presence or the absence of certain *MSTN* alleles, showed that this increase was due to effect the *MSTN*-E allelic variant, because the occurrence of both additional copy of the *MSTN*-A allele or one copy of the *MSTN*-E1 allele in the genotype of a lamb caused reduction in fore shank cut weight. Furthermore, we also found, that additional copy of *MSTN*-A was not advantageous in terms of its effect on weight of scrag and leg weight, whereas hind shank weight were higher due to the effect of the *MSTN*-E allele. Since other authors have performed their analyses on different lamb cuts (inter alia [17,37,38]) and they have investigated polymorphism in divergent parts of the *MSTN* gene than first intron, especially 3′UTR region (inter alia [15,34,38]), it is hard to directly compare our outcomes with their results. Worth mentioning is the fact, that similarly to present study, a large number of articles have been focused on non-coding regions of the *MSTN* gene, instead of its coding parts (inter alia [16,38]). For instance, Wang et al. [16], who investigated associations between SNPs in promoter region of the myostatin gene and carcass traits in New Zealand Romney sheep, observed significant effects of substitution in the c.-2449 position, but not in c.-2379, on loin yield and proportion. Also Hadjipavlou et al. [43] analyzed the g.-2429G>C SNP and they noticed its significant impact on muscle depth in commercial Charollais sheep. Similar effect was seen for the c.*1232G>A SNP in the 3′UTR region [43]. Complex analysis of associations between the c.*1232G>A SNP and wide spectrum of carcass traits were performed by Johnson et al. [38] in New Zealand Texel sheep. In general, based on bulk number of valuable measurements of carcass, e.g., total weights of lean, internal, and subcutaneous fat as well as bone weights in three lamb cuts, they confirmed that the A allele was associated with increased muscle and decreased fat mass. Additionally, they noticed that estimates for an additive effect were significant and were positive for muscle, but negative for fat traits [38]. Kijas et al. [15], who investigated not only the c.*1232G>A SNP, but also nucleotide substitutions in the promoter region and in the second intron of the *MSTN* gene, found that majority of significant effects of polymorphism in the myostatin gene on selected carcass traits were due to the substitution in the c.*1232 position in Australian sheep breeds. Furthermore, Haynes et al. [34] reported that homozygous AA lambs also in the c.*1232 position had higher dressing percentage, shortloin, topside, and round weights when compared to their counterparts with other genotypes in Australian sheep. They also confirmed higher lean and less fat mass in the AA homozygous lambs than in sheep with other genotypes in the c.*1232 position. Interestingly, Haynes et al. [34] found increased number of muscle fibers in the cross-section of LM between the 12th and 13th rib in lambs with AA genotype. In contrast, Masri et al. [35] did not show association between the c.*1232G>A SNP and several traits measured by video image analysis, i.e., weight of 5 lamb cuts both in Texel and Poll Dorset sired lambs. Worth mentioning is the fact that they observed significant effect of this SNP on several carcass traits describing area of different muscles, muscle proportion and total estimated muscle weight.

## 5. Conclusions

Polymorphisms in the *MSTN* gene are associated with increased muscle mass and decreased fatness [13,15]; therefore, we regarded this gene as genetic marker in selecting for better meat performance in Merino lambs. The importance of our present study lies in assessing the genotypic and allelic effects of the polymorphisms in the first intron of the *MSTN* gene on selected growth and slaughter traits in the Colored Polish Merino, the sheep breed, which is monomorphic in the c.*1232 position. A total of 7 growth and 16 carcass traits were investigated. The novelty in the present study was that 10 different meat cuts were separately subjected to association analysis. Significant genotypic effects of the polymorphisms in the first intron of the *MSTN* gene were found for BW2, loin and fore shank weights. Moreover, the *MSTN* alleles in the first intron of this gene significantly affected BW2, scrag, leg, fore and hind shank weights. Obtained results suggest that polymorphisms in the first intron of the *MSTN* gene are associated with abovementioned carcass traits and BW2 in Colored Polish Merino sheep; therefore, may be considered as genetic marker of carcass traits in this breed. Our results provide a foundation for further analysis of polymorphisms and association analyses of the *MSTN* gene with production traits as well gene expression study both in Merino and other sheep breeds worldwide, which regardless of monomorphism in the c.*1232 position, exhibit phenotypic variance in carcass traits.

## Figures and Tables

**Table 1:** Alleles identified in the first intron of the *MSTN* gene in Colored Polish Merino sheep.

Nucleotide Position	SNP	Allele			
		*MSTN*-A	*MSTN*-C	*MSTN*-E	*MSTN*-E1
c.373+18	G/T	G	T	T	T
c.373+101	C/T	C	C	C	T
c.373+240	T	T	T	T	T
c.373+241	T	T	T	T	T
c.373+243	A/G	G	A	G	G
c.373+246	T	T	T	T	T
c.373+249	C/T	T	C	T	T
c.373+259	G/T	G	T	T	T
c.373+323	C/T	C	T	C	C

**Table 2:** Allelic and genotypic frequencies in the first intron of the *MSTN* gene in Colored Polish Merino sheep.

Allele/Genotype (n = 264)		Frequency (%)
Allele	*MSTN*-A	87.7
	*MSTN*-C	2.3
	*MSTN*-E	5.5
	*MSTN*-E1	4.5
Genotype	*MSTN*-A/*MSTN*-A	75.4
	*MSTN*-A/*MSTN*-C	4.5
	*MSTN*-A/*MSTN*-E	11.0
	*MSTN*-A/*MSTN*-E1	9.1

**Table 3:** Results of the association analysis between the *MSTN* genotypes and growth traits (LSM ± Standard error) in Colored Polish Merino sheep.

Trait	Unit	LSM ^1^ ± Standard Error				*p*-Value
		*MSTN*-A/*MSTN*-A	*MSTN*-A/*MSTN*-C	*MSTN*-A/*MSTN*-E	*MSTN*-A/*MSTN*-E1	
n = 264		199	12	29	24	
Body weight at 2nd day of life	kg	**5.0 ± 0.08 ^a^**	**5.3 ± 0.22 ^a,b^**	**5.1 ± 0.14 ^a,b^**	**5.5 ± 0.18 ^b^**	**0.042**
Body weight at 30th day of life	kg	12.7 ± 0.17	12.9 ± 0.54	12.5 ± 0.35	13.1 ± 0.42	0.676
Body weight at 56th day of life	kg	19.2 ± 0.32	19.3 ± 0.81	19.2 ± 0.55	19.3 ± 0.69	0.999
Body weight at 78th day of life	kg	26.4 ± 0.48	24.9 ± 1.71	26.9 ± 0.76	26.7 ± 0.98	0.721
Average daily gain between 2nd and 30th day of life	g	256 ± 4.06	253 ± 14.48	246 ± 9.27	252 ± 10.41	0.808
Average daily gain between 30th and 56th day of life	g	249 ± 6.75	243 ± 15.40	258 ± 10.74	243 ± 13.33	0.686
Average daily gain between 56th and 78th day of life	g	329.8 ± 8.33	326.7 ± 23.30	319.1 ± 15.54	330.0 ± 19.21	0.914

^1^ LSM = Least squares mean; values with different superscripts (a, b) within a row are significantly different (*p* < 0.05). Values in bold were applied to highlight highly significant effect of the *MSTN* genotypes on growth traits.

**Table 4:** Results of the association analysis between a number of copies of the *MSTN* alleles and growth traits (LSM ± Standard error) in Colored Polish Merino sheep.

Trait	Unit	Allele	LSM ^1^ ± Standard Error				*p*-Value
			Number of Copies of the Allele				
			*MSTN*-A-1; *MSTN*-C, *MSTN*-E and *MSTN*-E1-0	n	*MSTN*-A-2; *MSTN*-C, *MSTN*-E and *MSTN*-E1-1	n	
Body weight at 2nd day of life	kg	*MSTN*-A	5.2 ± 0.12	65	5.1 ± 0.09	199	0.093
		*MSTN*-C	5.1 ± 0.09	252	5.3 ± 0.23	12	0.347
		*MSTN*-E	5.1 ± 0.09	235	5.06 ± 0.16	29	0.754
		*MSTN*-E1	**5.1 ± 0.07 ^a^**	240	**5.5 ± 0.18 ^b^**	24	**0.006**
Body weight at 30th day of life	kg	*MSTN*-A	12.4 ± 0.27	65	12.7 ± 0.18	199	0.996
		*MSTN*-C	12.7 ± 0.18	252	12.9 ± 0.54	12	0.709
		*MSTN*-E	12.8 ±0.18	235	12.5 ± 0.36	29	0.386
		*MSTN*-E1	12.7 ± 0.15	240	13.1 ± 0.41	24	0.298
Body weight at 56th day of life	kg	*MSTN*-A	19.2 ± 0.44	65	19.2 ± 0.32	199	0.961
		*MSTN*-C	19.2 ± 0.31	252	19.3 ± 0.80	12	0.937
		*MSTN*-E	19.1 ± 0.32	235	19.6 ± 0.58	29	0.415
		*MSTN*-E1	19.2 ± 0.31	240	19.3 ± 0.68	24	0.896
Body weight at 78th day of life	kg	*MSTN*-A	26.4 ± 0.59	65	26.5 ± 0.47	199	0.829
		*MSTN*-C	26.5 ± 0.46	252	26.6 ± 1.03	12	0.877
		*MSTN*-E	26.2 ± 0.44	235	27.2 ±0.77	29	0.263
		*MSTN*-E1	26.5 ± 0.46	240	26.5 ± 0.90	24	0.966
Average daily gain between 2nd and 30th day of life	g	*MSTN*-A	250 ± 6.43	65	256 ± 4.04	199	0.388
		*MSTN*-C	254 ± 3.68	252	253 ± 14.45	12	0.937
		*MSTN*-E	255 ± 3.79	235	246 ± 9.24	29	0.360
		*MSTN*-E1	254 ± 3.78	240	252 ± 10.38	24	0.822
Average daily gain between 30th and 56th day of life	g	*MSTN*-A	251 ± 8.71	65	249 ± 6.71	199	0.784
		*MSTN*-C	249 ± 6.43	252	242 ± 15.32	12	0.629
		*MSTN*-E	247 ± 6.61	235	265 ± 11.17	29	0.077
		*MSTN*-E1	250 ± 6.65	240	243 ± 13.33	24	0.593
Average daily gain between 56th and 78th day of life	g	*MSTN*-A	324 ± 11.88	65	330 ± 8.30	199	0.603
		*MSTN*-C	329 ± 7.95	252	327 ± 23.24	12	0.935
		*MSTN*-E	328 ± 7.68	235	332 ± 16.30	29	0.793
		*MSTN*-E1	328 ± 8.10	240	330 ± 19.21	24	0.918

^1^ LSM = Least squares mean; values with different superscripts (a, b) within a row are significantly different (*p* < 0.05). Values in bold were applied to highlight highly significant effect of a number of copies of the *MSTN* alleles on growth traits.

**Table 5:** Results of the association analysis between the *MSTN* genotypes and carcass traits (LSM ± Standard error) in Colored Polish Merino sheep.

Trait	Unit	LSM ^1^ ± Standard Error		*p*-Value
		*MSTN*-A/*MSTN*-A	*MSTN*-A/*MSTN*-E	
***n***		79	12	
**Carcass parts**				
Fore part of the carcass weight	g	2628 ± 13.23	2645 ± 29.98	0.599
Full loin part weight	g	1711 ± 10.96	1698 ± 24.18	0.539
Leg part weight	g	2217 ± 9.13	2239 ± 22.79	0.392
**Carcass cuts**				
Scrag weight	g	370 ± 5.25	328 ± 10.30	0.2671
Middle neck weight	g	526 ± 8.90	535 ± 15.18	0.535
Shoulder weight	g	1049 ± 5.24	1038 ± 11.93	0.387
Fore shank weight	g	**308 ± 2.03 ^a^**	**327 ± 5.27 ^b^**	**0.0018**
Breast and flank weight	g	1029 ± 8.08	1050 ± 19.77	0.329
Rib weight	g	473 ± 4.54	456 ± 10.04	0.104
Loin weight	g	**518 ± 4.73 ^a^**	**486 ± 12.44 ^b^**	**0.025**
Tenderloin weight	g	58 ± 0.95	60 ± 1.87	0.218
Leg weight	g	1815 ±9.10	1834 ± 20.47	0.365
Hind shank weight	g	401 ± 2.66	402 ± 6.31	0.844
**Leg**				
Muscle tissue yield	%	71.8 ± 0.30	71.7 ± 0.67	0.914
Fat tissue yield	%	12.4 ± 0.27	12.1 ± 0.62	0.655
Bone tissue yield	%	15.2 ± 0.17	15.7 ± 0.37	0.173

^1^ LSM = Least squares mean; values with different superscripts (a, b) within a row are significantly different (*p* < 0.05). Values in bold were applied to highlight highly significant effect of the *MSTN* genotypes on carcass traits.

**Table 6:** Results of the association analysis between a number of copies of the *MSTN* alleles and carcass traits (LSM ± Standard error) in Colored Polish Merino sheep.

Trait	Unit	Allele	LSM ^1^ ± Standard Error				*p*-Value
			Number of Copies of the Allele				
			*MSTN*-A-1; *MSTN*-E and *MSTN*-E1-0	n	*MSTN*-A-2; *MSTN*-E and *MSTN*-E1-1	n	
**Carcass parts**							
Fore part of the carcass weight	g	*MSTN*-A	2645 ± 21.56	25	2628 ± 12.60	79	0.491
		*MSTN*-E	2627 ± 12.25	92	2633 ± 26.52	14	0.871
		*MSTN*-E1	2624 ± 12.45	96	2624 ± 35.15	10	0.903
Full loin part weight	g	*MSTN*-A	1707 ± 17.48	25	1711 ± 10.33	79	0.824
		*MSTN*-E	1711 ± 9.89	92	1709 ± 21.43	14	0.947
		*MSTN*-E1	1708 ± 9.81	96	1740 ± 27.95	10	0.266
Leg part weight	g	*MSTN*-A	2255 ± 17.92	25	2214 ± 8.91	79	0.055
		*MSTN*-E	2219 ± 7.91	92	2228 ± 22.06	14	0.695
		*MSTN*-E1	2221 ± 8.01	96	2192 ± 24.95	10	0.266
**Carcass cuts**							
Scrag weight	kg	*MSTN*-A	**389 ± 7.53 ^a^**	25	**370 ± 4.65 ^b^**	79	**0.016**
		*MSTN*-E	371 ± 4.92	92	384 ± 9.43	14	0.207
		*MSTN*-E1	373 ± 5.58	96	363 ± 13.53	10	0.450
Middle neck weight	g	*MSTN*-A	535 ± 11.52	25	526 ± 7.72	79	0.429
		*MSTN*-E	526 ± 7.62	92	530 ± 13.39	14	0.759
		*MSTN*-E1	527 ± 8.39	96	510 ± 19.05	10	0.367
Shoulder weight	g	*MSTN*-A	1054 ± 11.90	25	1051 ± 7.09	79	0.811
		*MSTN*-E	1053 ± 6.05	92	1040 ± 13.90	14	0.404
		*MSTN*-E1	1051 ± 6.06	96	1044 ± 17.94	10	0.674
Fore shank weight	g	*MSTN*-A	**320 ± 3.82 ^a^**	25	**308 ± 2.10 ^b^**	79	**0.005**
		*MSTN*-E	**308 ± 2.07 ^a^**	92	**322 ± 4.91 ^b^**	14	**0.009**
		*MSTN*-E1	311 ± 2.31	96	299 ± 7.87	10	0.147
Breast and flank weight	g	*MSTN*-A	1049 ± 14.25	25	1031 ± 8.02	79	0.268
		*MSTN*-E	1032 ± 7.65	92	1050 ± 17.70	14	0.340
		*MSTN*-E1	1034 ± 7.22	96	1045 ± 22.07	10	0.637
Rib weight	g	*MSTN*-A	462 ± 6.88	25	471 ± 4.01	79	0.223
		*MSTN*-E	470 ± 3.86	92	455 ± 8.72	14	0.100
		*MSTN*-E1	469 ± 3.79	96	460 ± 11.16	10	0.483
Loin weight	g	*MSTN*-A	495 ± 9.61	25	516 ± 5.26	79	0.053
		*MSTN*-E	515 ± 5.05	92	500 ± 12.39	14	0.279
		*MSTN*-E1	510 ± 4.40	96	537 ± 14.42	10	0.083
Tenderloin weight	g	*MSTN*-A	60 ± 1.38	25	58 ± 0.85	79	0.114
		*MSTN*-E	58 ± 0.77	92	58 ± 1.64	14	0.837
		*MSTN*-E1	58 ± 0.77	96	56 ± 2.51	10	0.359
Leg weight	g	*MSTN*-A	**1856 ± 16.45 ^a^**	25	**1813 ± 8.75 ^b^**	79	**0.020**
		*MSTN*-E	1817 ± 8.93	92	1820 ± 18.95	14	0.899
		*MSTN*-E1	1820 ± 8.35	96	1798 ± 24.17	10	0.372
Hind shank weight	g	*MSTN*-A	397 ± 4.99	25	401 ± 3.04	79	0.499
		*MSTN*-E	**399 ± 2.90 ^a^**	92	**414 ± 6.30 ^b^**	14	**0.027**
		*MSTN*-E1	401 ± 2.99	96	415 ± 9.83	10	0.155
**Leg**							
Muscle tissue yield	%	*MSTN*-A	71.6 ± 0.49	25	71.8 ± 0.29	79	0.752
		*MSTN*-E	71.8 ± 0.29	92	71.6 ± 0.60	14	0.753
		*MSTN*-E1	71.8 ± 0.27	96	71.2 ± 0.78	10	0.457
Fat tissue yield	%	*MSTN*-A	12.8 ± 0.56	25	12.4 ± 0.25	79	0.510
		*MSTN*-E	12.6 ± 0.28	92	12.3 ± 0.59	14	0.664
		*MSTN*-E1	12.5 ± 0.25	96	13.7 ± 0.73	10	0.094
Bone tissue yield	%	*MSTN*-A	15.1 ± 0.28	25	15.2 ± 0.16	79	0.690
		*MSTN*-E	15.1 ± 0.15	92	15.7 ± 0.34	14	0.123
		*MSTN*-E1	15.2 ± 0.15	96	14.6 ± 0.44	10	0.151

^1^ LSM = Least Squares Mean; values with different superscripts (a, b) within a row are significantly different (*p* < 0.05). Values in bold were applied to highlight highly significant effect of a number of copies of the *MSTN* alleles on carcass traits.
